# Association of VEGF and VDR gene- gene and gene- smoking interaction on risk of multiple myeloma in Chinese Han population

**DOI:** 10.18632/oncotarget.16510

**Published:** 2017-03-23

**Authors:** Peng Chen, Zhen-Lan Du, Yuan Zhang, Bing Liu, Zhi Guo, Jin-Xing Lou, Xue-Peng He, Hui-Ren Chen

**Affiliations:** ^1^ Department of Hematology, PLA Army General Hospital, Beijing, 100700, People's Republic of China; ^2^ Department of Hematology and Oncology, Affiliated Bayi Children's Hospital, PLA Army General Hospital, Beijing, 100700, People's Republic of China; ^3^ National Engineering Laboratory for Birth Defects Prevention and Control of Key Technology, Beijing, 100700, People's Republic of China; ^4^ Beijing Key Laboratory of Pediatric Organ Failure, Beijing, 100700, People's Republic of China

**Keywords:** multiple myeloma, vascular endothelial growth factor, vitamin D receptor, smoking, interaction

## Abstract

**Aims:**

To investigate the association of several single nucleotide polymorphisms (SNPs) within vascular endothelial growth factor (VEGF) and vitamin D receptor (VDR) gene polymorphisms and additional gene- gene and gene- smoking interaction with multiple myeloma (MM) risk in Chinese population.

**Methods:**

Generalized multifactor dimensionality reduction (GMDR) was used to screen the best interaction combination among SNPs and smoking. Logistic regression was performed to investigate association between 6 SNPs within VEGF and VDR gene, additional gene- gene and gene- smoking interaction on MM risk.

**Results:**

MM risk is significantly higher in carriers with the rs699947- A allele within VEGF gene than those with CC genotype (CA+ AA versus CC), adjusted OR (95%CI) =1.72 (1.19-2.33), and higher in carriers with rs2228570- T allele within *VDR* gene than those with CC genotype (CT+ TT versus CC), adjusted OR (95%CI) = 1.68 (1.26-2.17). We also found a significant two-locus model (p=0.0010) involving rs699947 and rs2228570, and a significant two-locus model (p=0.0107) involving rs2228570 andsmoking. Participants with rs699947- CA+AA and rs2228570- CT+TT genotype had the highest MM risk, compared to participants with rs699947- CC and rs2228570- CC genotype, OR (95%CI) = 3.12 (1.82 -4.61). Smokers with rs2228570- CT+TT genotype had the highest MM risk, compared to never- smokers with rs2228570- CC genotype, OR (95%CI) = 3.27 (1.74-4.86).

**Conclusions:**

We found that the A allele of rs699947 within VEGF and T allele of rs2228570 within *VDR* gene, interaction between rs699947 and rs2228570, rs2228570 andsmoking were all associated with increased MM risk.

## INTRODUCTION

Multiple myeloma (MM) is a fatal disorder of plasma cells derived from an early precursor of B-cell lineage and is responsible for about 2% of all cancer deaths [1]. The etiology of MM is not well studied until now. Previous evidences suggested that some environmental factors including exposure to radiation, herbicides, insecticides, benzene, and other organic solvents may be risk factors for MM [2]. Recent studies indicated that BMI [3] and alcohol drinking [4] were associated with MM risk. On the other hand, racial differences for MM incidence and familial clusters of the disease indicated that the role of genetic predisposition to MM should be taken seriously [2].

Recent years, some genes related to MM susceptibility have been reported in some studies, including vascular endothelial growth factor (VEGF) gene and vitamin D receptor (VDR) polymorphisms. The VEGF gene is located on chromosome 6p21.3 and consists of 8 exons exhibiting alternate splicing to form a family of proteins [5]. Activation of the VEGF receptors could increase the production of various haematopoietic growth factors, and then stimulate the myeloma cell in a paracrine pathway [6, 7]. The expression of VEGF in response to different stimuli is highly variable among individuals. Single nucleotide polymorphisms (SNPs) in the VEGF gene may contribute to this variation [8]. Some polymorphisms within VEGF have been reported association with some types of cancer, such as rs699947, rs2010963 and rs833061 and so on [9–11]. However, the association between VEGF gene and MM risk were not well verified [12–14].

Vitamin D is a fundamental mediator of skeletal metabolism due to its ability to regulate calcium homeostasis. Its deficiency negatively affects bone mineralization and stimulates osteoclast-mediated bone resorption [15]. Studies have reported low serum vitamin D as an important contributor to the skeletal complications which was a major cause of morbidity in MM [16, 17]. 1, 25(OH) _2_ D3 was the hormonal derivative of vitamin D and have growth-regulatory effect through binding to the intranuclear receptor vitamin D receptor (VDR) [18]. Several polymorphisms have been identified in various introns and exons of VDR gene, including ApaI, BsmI and FokI [19], and recent studies have indicated that these SNPs may be linked with many kinds of cancer risks [20–22]. But the association between VDR gene and MM risk were less studied. Smoking was a risk factor for many types of cancer, but to date, no study focused on gene- smoking interaction on MM risk. In consideration of the limited study focused on association of VEGF and VDR gene with MM risk, and these studies concluded inconsistent results. So in current study, we aimed to investigate the association of several SNPs within VEGF and VDR gene and additional gene- gene and gene- smoking interaction with MM risk in Chinese population.

## RESULTS

A total of 1388 participants (733 males, 655 females) were selected, including 460 MM patients and 928 control participants. The mean age of all participants was 62.3 ± 14.2 years. Participants characteristics stratified by cases and controls were shown in Table 1. The mean of age and distributions of males and alcohol drinkers were not significantly different between cases and controls. The mean of BMI was higher in controls than cases. The rate of smokers was higher in cases than controls.

**Table 1:** General characteristics of 1388 study participants in case and control group

Variables	Case group(n=460)	Control group(n=928)	*p-*values
Age (year) (Means± SD)	62.6±14.5	61.9±15.8	0.425
Males, N (%)	248 (53.9)	485 (52.3)	0.562
Smokers, N (%)	201 (43.7)	257 (27.7)	<0.001
Alcohol drinkers, N (%)	139 (30.2)	246 (26.5)	0.146
BMI (kg/m^2^) (Means± SD)	23.1±9.8	24.6±9.2	0.005
Family history of MM N (%)	43 (9.3)		
Staging			
DS I–II	108 (23.5)		
DS III	352 (76.5)		
ISS I–II	276 (60.0)		
ISS III	184 (40.0)		
Percentage of bone marrow plasma cells (range)	35 (14–93)		
Creatinine in mg/dl (range)	1.7 (0.5–6.1)		
C-reactive protein in mg/dl (range)	0.6 (0.1–15.8)		

All genotypes are distributed according to HWE in controls (all *p* values are more than 0.05). The frequencies for the rs699947- A allele within VEGF gene and rs2228570- T allele within *VDR* gene were significantly higher in MM cases than control group (31.2% *vs*20.3%, 28.8% *vs*19.5%). Logistic regression analysis showed that MM risk was significantly higher in carriers with the rs699947- A allele within VEGF gene than those with CC genotype (CA+ AA versus CC), adjusted OR (95%CI) =1.72 (1.19-2.33), and higher in carriers with the rs2228570- T allele within *VDR* gene than those with CC genotype (CT+ TT versus CC), adjusted OR (95%CI) = 1.68 (1.26-2.17). However, we did not find any significant association of the others SNPs in VEGF and VDR gene with MM risk after covariates adjustment. (Table 2)

**Table 2:** Genotype and allele frequencies of 6 SNPs between case and control group

SNP	Genotypes andAlleles	Frequencies N (%)		OR(95%CI)*	HWE test forcontrols
		Control (n=928)	Case (n=460)		
VEGF gene					
rs2010963(+405 G>C)					
	Co-dominant				
	GG	542(58.4)	241 (52.4)	1.00 (ref)	0.935
	GC	335(36.1)	181 (39.3)	1.28(0.83-1.87)	
	CC	51(5.5)	38 (8.3)	1.51(0.79-2.34)	
	Dominant				
	GG	542(58.4)	241 (52.4)	1.00 (ref)	
	GC+CC	386(41.6)	219(47.6)	1.36(0.81-1.96)	
	Allele, C (%)	437(23.5)	257(27.9)		
rs699947(2578 C>A)					
	Co-dominant				
	CC	598(64.4)	229(49.8)	1.00 (ref)	0.108
	CA	284(30.6)	175(38.0)	1.36(1.08-1.72)	
	AA	46 (5.0)	56(12.2)	2.38(1.59-3.17)	
	Dominant				
	CC	598(64.4)	229(49.8)	1.00 (ref)	
	CA+AA	330(35.6)	231(50.2)	1.72(1.19-2.33)	
	Allele, A (%)	376(20.3)	287(31.2)		
rs833061(460 C>T)					
	Co-dominant				
	CC	532(57.3)	236 (51.3)	1.00 (ref)	0.105
	CT	329(35.5)	177(38.5)	1.24 (0.81-1.80)	
	TT	67 (7.2)	47(10.2)	1.48 (0.86-2.13)	
	Dominant				
	CC	532(57.3)	236 (51.3)	1.00 (ref)	
	CT+TT	396(42.7)	224(48.7)	1.30(0.83-1.91)	
	Allele, T (%)	463(25.0)	271(29.5)		
VDR gene					
ApaI (rs7975232)					
	Co-dominant				
	GG	537(57.9)	238 (51.7)	1.00 (ref)	0.288
	GT	330(35.6)	175 (38.1)	1.24(0.81-1.83)	
	TT	61(6.6)	47 (10.2)	1.57(0.75-2.40)	
	Dominant				
	GG	537(57.9)	238 (51.7)	1.00 (ref)	
	GT+TT	391(42.1)	222(48.3)	1.26(0.79-1.99)	
	Allele, T (%)	452(24.4)	269(29.2)		
FokI (rs2228570)					
	Co-dominant				
	CC	607 (65.4)	237 (51.5)	1.00 (ref)	0.234
	CT	280 (30.2)	181 (39.3)	1.47 (1.22-1.85)	
	TT	41 (4.4)	42 (9.2)	2.12 (1.43-2.88)	
	Dominant				
	CC	607 (65.4)	237 (51.5)	1.00 (ref)	
	CT+TT	321 (34.6)	223 (48.5)	1.68 (1.26-2.17)	
	Allele, T (%)	362 (19.5)	265 (28.8)		
BsmI (rs1544410)					
	Co-dominant				
	AA	590 (63.6)	259(56.3)	1.00 (ref)	
	AG	290 (31.2)	161 (35.0)	1.32 (0.92-1.81)	
	GG	48 (5.2)	40 (8.7)	1.52 (0.83-2.35)	
	Dominant				
	AA	590 (63.6)	259(56.3)	1.00 (ref)	
	AG+GG	338 (36.4)	201 (43.7)	1.38 (0.90-1.87)	
	Allele, G (%)	386 (20.8)	241 (26.2)		

* Adjusted for gender, age, smoking and alcohol status, BMI and WC.

Table 3 summarized the results obtained from GMDR analysis. We found a significant two-locus model (p=0.0010) involving rs699947 and rs2228570, indicating a potential interaction between rs699947 and rs2228570 on MM risk. Overall, the cross-validation consistency of this two- locus model was 9/ 10, and the testing accuracy was 60.72%. We also found a significant two-locus model (p=0.0107) involving rs2228570 and smoking, indicating a potential interaction between rs2228570 and smoking on MM risk, Overall, the cross-validation consistency of this two- locus model was 10/ 10, and the testing accuracy was 60.11%.

**Table 3:** GMDR analysis on the best gene–gene and gene- smoking interaction models

Locus no.	Best combination	Cross-validationconsistency	Testingaccuracy	*p-values*
Gene- gene interactions*				
2	rs699947 rs2228570	9/10	0.6072	0.0010
3	rs699947 rs2228570 rs2010963	8/10	0.5399	0.1719
4	rs699947 rs2228570 rs2010963 rs1544410	7/10	0.5399	0.3770
5	rs699947 rs2228570 rs2010963 rs1544410 rs833061	6/10	0.4958	0.4258
6	rs699947 rs2228570 rs2010963 rs1544410 rs833061 rs7975232	5/10	0.4958	0.6230
Gene- smoking interactions ^*^				
2	rs2228570 Smoking	10/10	0.6011	0.0107
3	rs2228570 rs699947 Smoking	9/10	0.5399	0.1719
4	rs2228570 rs699947 rs2010963 Smoking	8/10	0.4958	0.3770
5	rs2228570 rs699947 rs2010963 rs1544410 Smoking	7/10	0.4958	0.4258
6	rs2228570 rs699947 rs2010963 rs1544410 rs833061	6/10	0.4958	0.6230
7	rs2228570 rs699947 rs2010963 rs1544410 rs833061 rs7975232 Smoking	5/10	0.4958	0.9893

* Adjusted for gender, age, smoking, drinking and BMI for gene- gene interaction analysis

**Adjusted for gender, age, drinking and BMI for gene- smoking interaction analysis

In the stratified logistic regression, we found that participants with rs699947- CA+AA within VEGF gene and rs2228570- CT+TT genotype within VDR gene had the highest MM risk, compared to participants with rs699947- CC within VEGF gene and rs2228570- CC genotype, OR (95%CI) = 3.12 (1.82 -4.61), after covariates adjustment. We also found that current smokers with rs2228570- CT+TT genotype had the highest MM risk, compared to never- smokers with rs2228570- CC within VDR genotype, OR (95%CI) = 3.27 (1.74-4.86), after covariates adjustment (Table 4).

**Table 4:** Analysis for gene-gene and gene- smoking interaction by using logistic regression

Variable 1	Variable 2	OR (95% CI)*	*P-values*
rs699947	rs2228570		
CC	CC	1.00	-
CA+AA	CC	1.48 (1.14 -1.92)	0.001
CC	CT+TT	1.38 (1.05-1.79)	0.032
CA+AA	CT+TT	3.12 (1.82 -4.61)	<0.001
rs2228570	Smoking		
CC	Never	1.00	-
CT+TT	Never	1.35(1.04 -1.82)	0.038
CC	Current	1.56 (1.17-2.01)	0.002
CT+TT	Current	3.27 (1.74-4.86)	<0.001

* Adjusted for gender, age, drinking and BMI

## DISCUSSION

In this study, we found that both the A allele of rs699947 within VEGF and T allele of rs2228570 within *VDR* gene are significantly associated with increased in MM cases. The VEGF gene is located on chromosome 6p21.3 and consists of 8 exons exhibiting alternate splicing to form a family of proteins[4]. Some studies [9–11] have reported that several SNPs within VEGF were associated with some types of cancer, such as oral, breast, glioma, colorectal and lung. However, the association between VEGF gene and MM risk has not been interpreted so far, and less studies focused on this relationship previously [12–14]. Andersen et al [12] suggested that the VEGF haplotype ACG may influence the efficacy of thalidomide treatment in patients with multiple myeloma, although they did not find any association between SNP and MM risk. Another study [13] also did not find relationship between SNP within VEGF gene and MM risk, however they indicated that VEGF and GST genotypes can combine to influence the risk for MM in south-eastern Brazil. Brito et al [14] also suggested that inherited abnormalities in VEGF (−2578C/A, −1154G/A, −634G/C) pathways influence the risk and aggressiveness of MM, relatively small sample size may be the main reason for the negative result obtained in these studies. Berardi [23] et al firstly indicated that inherited abnormalities in VEGFR-3 pathways influence the risk and aggressiveness of thymic epithelial tumors (TETs).

VDR is encoded by a large gene (>100 kb) located on chromosome 12cen-q12 containing 14 exons. BsmI (rs1544410), ApaI (rs7975232) and FokI (rs2228570) were three common SNPs in VDR gene polymorphism. Studies have reported low serum vitamin D as an important contributor to the skeletal complications which is a major cause of morbidity in MM [16, 17]. Several polymorphisms have been identified in various introns and exons of VDR gene most commonly ApaI, BsmI and FokI [19], and recent studies [20–22] have indicated that these SNPs may be linked with many kinds of cancer risks. To date, just one population based study [24] was conducted to investigate the association between VDR SNPs and MM risk. In this study, the authors indicated that the FokI polymorphism was associated increased susceptibility to MM in the ethnic Kashmiri population.

MM susceptibility was influenced by both genetic and environment factors, and previously several environmental factors associated with MM were reported, including exposure to radiation, herbicides, insecticides, benzene and other organic solvents may be risk factors for MM [2]. Cigarette smoking has been suggested to play a crucial role in increasing the risk of some types of cancer risk, but the association with MM risk was inconclusive [25, 26]. In current study, we found that the smoking rate was higher in MM cases than controls, it means that smoking may be positively associated with MM risk, so in this study we investigated not only gene- gene interaction, but also gene- environment interaction between SNPs and smoking. We found a potential interaction between rs699947 and rs2228570 and a potential interaction between rs2228570 andsmoking on MM risk, participants with rs699947- CA+AA within VEGF gene and rs2228570- CT+TT genotype within VDR gene had the highest MM risk, compared to participants with rs699947- CC within VEGF gene and rs2228570- CC genotype, in addition, current smokers with rs2228570- CT+TT genotype also had the highest MM risk, compared to never- smokers with rs2228570- CC within VDR genotype.

There several limitations in our study. Firstly, more SNPs within VEGF and VDR gene should been included in the analysis, not only for just 6 SNPs. Secondly, some others environmental risk factors should be investigated in the gene- environment interaction analysis. Thirdly, the results obtained from our study should be checked in the future studies. In conclusion, we found that the A allele of rs699947 within VEGF and T allele of rs2228570 within *VDR* gene, interaction between rs699947 and rs2228570, rs2228570 andsmoking were all associated with increased MM risk.

## MATERIALS AND METHODS

### Subjects

All study participants were recruited between June 2008 and March 2015 from the PLA Army General Hospital. The diagnostic criteria for MM have been described elsewhere and the clinical staging system for MM that was used according to International Myeloma Working Group criteria [27]. Controls were randomly selected from healthy volunteers from community-based chronic non-communicable diseases screening program conducted with a 1:2 matched (age and sex) in the same region. Consequently, a total of 460 newly diagnosed MM cases and 928 control participants were included in current study. The mean age of all participants is 62.3 ± 14.2 years. Questionnaire investigation was conducted for all participants, and data on demographic information, clinical and biochemical index for all participants were obtained. Body weight and height were measured. Body mass index (BMI) was calculated as weight in kilograms divided by the square of the height in meters. Current cigarette smokers were those who self-reported smoking cigarettes at least once a day for 1 year or more. Alcohol consumption was expressed as the sum of milliliters of alcohol per week from wine, beer, and spirits. Blood samples were collected from each participant in the morning after at least 8 hours of fasting. Informed consent was obtained from all participants.

### Genomic DNA extraction and genotyping

The SNPs were selected based on the NCBI database (http://www.ncbi.nlm.nih.gov/projects/SNP) according to the following three criteria: 1) located in a gene fragment that could have functional effects, and 2) with a minor allele frequency (MAF) of at least 5%, 3) previously reported associations with any type of cancer. Taking into account the limited human resources and financial resources, a total of 3 SNPs within VEGF and 3 SNPs within VDR gene were selected for genotyping, including: rs699947, rs2010963 and rs833061 within VEGF gene, rs2228570, rs1544410 and rs7975232 within VDR gene. Genomic DNA from participants was extracted from EDTA-treated whole blood, using the DNA Blood Mini Kit (Qiagen, Hilden, Germany) according to the manufacturer's instructions and stored at -20°C until use. The genotypes of six SNPs were detected by polymerase chain reaction-restriction fragment length polymorphism (PCR–RFLP) method. The nucleotide sequence of primers and description for the 6 SNPs were shown in Table 5. Genotyping for VEGF SNPs: In each 0.20-mL PCR reaction tube, 1 μL of genomic DNA (100 ng/mL), 1 μL of each primer (10 μM), 10 μL of 2X Prime Taq Premix, and appropriate amount of ddH_2_O were added. The PCR-cycling conditions: 5 minutes at 95°C, followed by 30 cycles of 30 seconds at 95°C, 30 seconds at 62°C for rs2010936, 63°C for rs3025039 and rs833061, 61°C for rs699947, 60°C for rs35569394, and 30 seconds at 72°C with a final extension step of 72°C for 5 min. Genotyping for VDR SNPs: The reaction volume was 25 mL, containing a Mastermix containing Taq DNA polymerase, dNTPs mix and reaction buffer (Shanghai, China). A total of 0.5μM of each primer, 2μl of template cDNA, 9μl 2.5* Mastermix, and 8.5μl distilled water, (ddH_2_O) were used in all reactions (in triplicate). Amplification and detection were carried out as follows. The protocol (denaturation 95°C, 15 s; annealing 60°C, 60 s; elongation 72°C, 45 s) consisted of 40 cycles to ensure a linear range of the PCR reaction.

**Table 5:** Description and probe sequence for 6 SNPs used for PCR analysis

SNP ID	Chromosome	FunctionalConsequence	Restrictionenzyme	Nucleotidesubstitution	Primer sequences
**VEGF gene**					
rs2010963+405 G>C	6:43770613	Upstream variant 2KB, utr variant 5 prime	FaqI	G > C	F:5′-TTGCTTGCCATTCCCCA CTTGA-3′R: 5′-CCGAAGCGAGAACAGC CCAGA-3′
rs833061460 C>T	6:43769749	Upstream variant 2KB	HinpI	C>T	F:5′-TGAGTGTGTGCGTGTGGG GTTGAGCG-3′R: 5′-AGAGCCGTTCCCTCTTT GCTAG-3′
rs6999472578 C>A	6:43768652	Upstream variant 2KB	BstyI	C>A	F:5′-GGCCTTAGGACACCATACC-3′R: 5′-CACAGCTTCTCCCCTATCC-3′
**VDR gene**					
FokI (rs2228570)	12:47879112	Missense	FokI	C>T	Forward: GCACTGACTCTGGC TCTGACReverse: ACCCTCCTGCTCCT GTGGCT
BsmI (rs1544410)	12:47846052	Intron variant, upstream variant 2KB	FspI	A>G	Forward: GGAGACACAGATAA GGAAATACReverse: CCGCAAGAAACCTCAA ATAACA
ApaI (rs7975232)	12:47845054	Intron variant, upstream variant 2KB	ApaI	G>T	Forward: TGGGCACGGGGATAG AGAAGReverse: ACGGAGAAGTCACTG GAGGG

Genotyping results were confirmed by randomly assaying 10% of the original specimens for replication to exclude genotyping errors. There were no discrepancies between genotypes determined in duplicate.

### Statistical analysis

All statistical analyses were performed using the SPSS 22.0 software package (SPSS Inc, Chicago) for Windows 7. The means and SDs were calculated for normally distributed continuous variables and were compared by Student's t test, and percentages were calculated for categorical variables and were analyzed using χ^2^ test. Departure from Hardy-Weinberg equilibrium (HWE) in controls was tested using SNPstats (http://bioinfo.iconcologia.net/SNPstats). Generalized multifactor dimensionality reduction (GMDR) was used to screen the best interaction combination among SNPs and smoking. Logistic regression was performed to investigate association of 6 SNPs within VEGF and VDR gene, additional gene- gene and gene- smoking interaction with MM risk. All reported *p*-values were two-tailed, and those less than 0.05 were considered statistically significant.
